# A Rare Coexistence of Retroperitoneal Pararenal Castleman's Disease with Focal Nodular Hyperplasia

**DOI:** 10.1155/2013/537593

**Published:** 2013-03-04

**Authors:** Theodosios Theodosopoulos, Andreas Karakatsanis, Anneza I. Yiallourou, Fotios Nikolakopoulos, Vassiliki Psychogiou, Eleni Karvouni, Dionysios Voros

**Affiliations:** ^1^2nd Department of Surgery, Aretaieion Hospital, University of Athens, 76 Vasilissis Sophias Avenue, 11528 Athens, Greece; ^2^Department of Pathology, Aretaieion Hospital, University of Athens, 76 Vasilissis Sophias Avenue, 11528 Athens, Greece

## Abstract

Castleman's disease is a distinct form of lymph node hyperplasia divided into a solitary and a multicentric type. The solitary type occurs most commonly in the mediastinum and is usually asymptomatic. We present a patient with Castleman's disease of the hyaline-vascular solitary type located in the retroperitoneum. The patient was a 38-year-old male, who presented to our hospital with fever. The imaging workup revealed a retroperitoneal mass, measuring 4 × 6 cm, located lateral to the aorta, inferior to the left renal artery and vein, and posterior to the left testicular vein. At workup, a solid hepatic lesion, 3 cm in diameter, located in the left lobe of the liver, segment IV, was also identified. Both lesions were surgically excised. The retroperitoneal tumor had the features of angiofollicular hyperplasia (Castleman's disease), hyaline-vascular type, whereas a diagnosis of focal nodular hyperplasia was made for the hepatic lesion. The patient is well at fourty months followup postoperatively. Surgical excision is the treatment of choice for unifocal Castleman's disease.

## 1. Introduction

Castleman's disease is a rare form of lymph node hyperplasia of unknown etiology, which has been divided into a solitary and a multicentric type. The solitary type is located mostly in the mediastinum, but it may also occur in other anatomical sites. It is usually asymptomatic. Occasionally it may cause symptoms from pressure of adjacent organs. The multicentric type of the disease is usually symptomatic, with severe systematic phenomena and may be associated with lymphoma, myeloma, and Kaposi's sarcoma. We present a rare case of solitary retroperitoneal Castleman's disease coexisting with focal nodular hyperplasia of the liver in an otherwise asymptomatic patient.

## 2. Case Presentation

**Figure 1:**
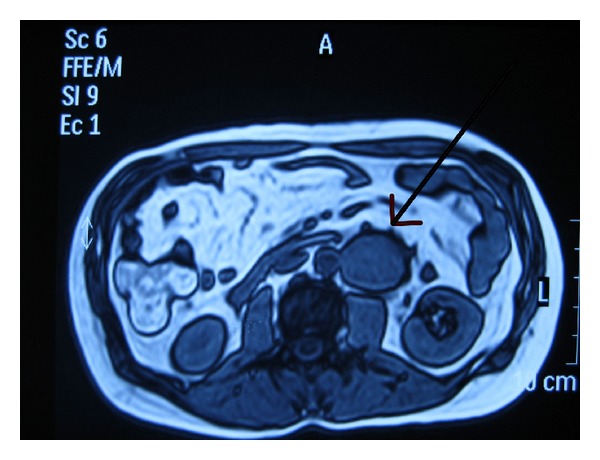
Abdominal MRI imaging depicting the lesion.

A 38-year-old man with unremarkable medical history presented with fever. Clinical workup included an abdominal ultrasound scan which revealed a mass of 6,5 × 5 cm located in the left retroperitoneum and a lesion of 3,3 cm in diameter in the left lobe of the liver. The retroperitoneal mass was solid and was in contact with the left renal vessels, without any hemodynamic features of malignancy. The most probable imaging diagnosis was a teratoma. The hepatic lesion was solid and hypoechoic, with increased vascularization, most likely presenting focal nodular hyperplasia. No other findings were present. Consequently, an abdominal CT scan was performed to delineate the retroperitoneal pararenal mass and an abdominal MRI (Figure 1) for the investigation of the hepatic lesion, which verified the findings mentioned above, without, however pointing in favor of any particular diagnosis.

Physical examination, routine blood count, and blood biochemistry were normal. In particular, no tumor markers (CEA, CA19-9, and AFP) were detected and the viral markers (HIV-1, HIV-2, HBV, and HCV) were negative. The chest X-ray was unremarkable. 

**Figure 2:**
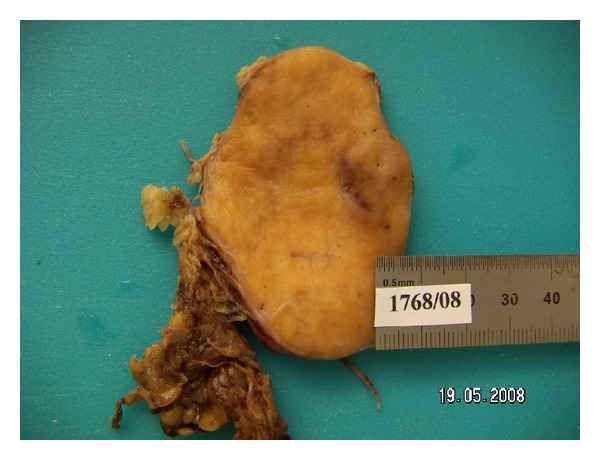
Retroperitoneal pararenal Castleman's disease.

**Figure 3:**
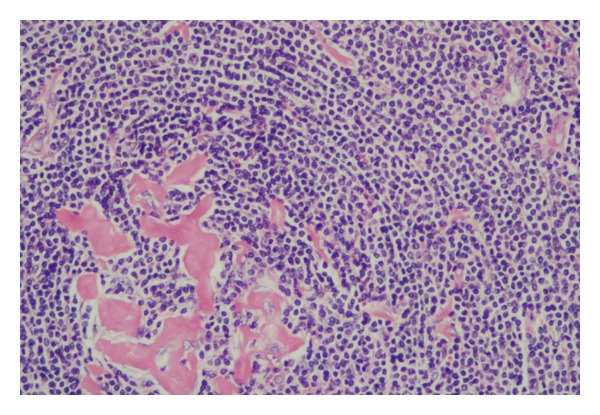
Hyaline-vascular Castleman's disease.

At laparotomy, a retroperitoneal tumor of 7 × 5 × 4 cm (Figure 2) was discovered and excised *en bloc* with the peri-renal fat and sent to the pathology department for intraoperative evaluation. The frozen section examination identified a lymphoid lesion and the definite diagnosis was deferred. Subsequently, the patient underwent wedge resection for the hepatic lesion. Histology confirmed the diagnosis of focal nodular hyperplasia. Paraffin sections of the retroperitoneal tumor revealed angiofollicular hyperplasia (Castleman's disease) of the hyaline-vascular type (Figure 3). In the fibrofatty tissue that was attached to the tumor a few lymph nodes were grossly identified. Histologic examination of the excised lymph nodes was unremarkable. The patient had an uneventful postoperative course and was discharged on the seventh postoperative day. Laboratory evaluation and imaging studies to exclude the presence of other malignancy were unremarkable, and the patient is disease free at fourty months of followup.

## 3. Discussion

Castleman's disease represents a distinct type of lymph node hyperplasia, rather than a neoplasm or a hamartoma. Its etiology is unknown and was first described by Symmers in 1921 [1], but it was characterized by Castleman et al. in 1956 as a benign lymph node hyperplasia [2]. It is usually seen in adults, the majority of which are under the age of 30, but it can also present in children. The disease is usually located in the mediastinum, where it was firstly described by Castleman, but it can also be found in other lymph node areas, such as the axilla, the neck, the abdomen, and the retroperitoneal space. It can also occur in the soft tissue of the extremities [3–6], whereas less common locations of the disease involve extranodal tissue as the epidural space, the pleura, the lungs, the breast, the adrenal gland, and the pancreas [3, 5, 6]. However, a pararenal retroperitoneal location of Castleman's disease is uncommon. According to a review by Testa et al. [6], pararenal location of Castleman's disease is particularly rare, accounting for only 2% of all cases. Abdominal location was found in 12% of the cases reported. 

 There are 2 different histologic subtypes of Castleman's disease as identified by Keller et al. in 1972 [3], each one with distinct clinical findings, prognosis, and treatment: the hyaline-vascular type (85–90%) and the plasma cell type. It has been shown, however, that these two different subtypes are not always clearly separated, and that mixed types can also occur.

 The hyaline-vascular type is the most common (90%). It is usually unifocal, forming a mass up to 15 cm. It is usually asymptomatic and identified as an incidental finding in U/S or CT scans. When it is large, it may cause symptoms, such as cough, dyspnea, neuropathy, jaundice, postprandial discomfort, anorexia, vomiting, and urinary retention [7] due to pressure of adjacent organs. It usually appears in young (mainly 3rd decade), otherwise healthy patients. Histologically the lymph node is replaced by follicles with hyalinization of the wall of the follicular center arterioles and prominent concentric whirls of mantle lymphocytes, in an onion skin feature. The sinuses are absent [3, 4]. Surgical resection is the standard treatment, with an excellent prognosis (almost 100% five-year survival after resection) whereas recurrence after treatment is extremely rare and is always related to incomplete excision of the lesion [4, 8, 9]. The unifocal type of Castleman's disease is closely related (>90%) to the hyalinovascular type of the disease [4, 6, 7]. The remaining percentage is related to the plasma cell type and, with the exception of the aforementioned symptoms, there is also anorexia, weight loss, night sweats, and severe fatigue, anemia, hypoalbuminemia, and hypergammaglobulinemia as consequence of elevated expression of IL-6 [10].

 The plasma cell type (10%) is usually associated with the multifocal form of the disease [4, 6, 7]. Histologically, it has the same appearance with the hyaline-vascular type with the additional feature of sheets of plasma cells in the interfollicular areas [3, 4]. It presents in older patients (6th decade). It is usually accompanied by systemic symptoms such an anorexia, weight loss, night sweats, and severe fatigue. Anemia, hypoalbuminemia, and hyperglobulinemia are also usually present [10]. There may be lymphadenopathy, mild hepatosplenomegaly, edema, or peripheral neuropathy. The disease is persistent and the main symptoms come from the lungs and the kidneys (dyspnea and renal failure). There is an association to lymphoma, myeloma, or Kaposi sarcoma, whereas there is close relation with other autoimmune and paraneoplastic phenomena (such as paraneoplastic, amyloidosis, as well as an overlap with POEMS syndrome (polyneuropathy, organomegaly, endocrinopathy, M-protein, skin changes) [7]. There is also a clear association with the viral status of the patient, especially HHV-8 and HIV [8, 9, 11]. The etiology of the plasma cell type of Castleman's disease is related to chronic Human Herpes virus 8 (HSV8), as HSV8 has been found in lymphoid cells in case of the plasma cell type, with the most probable hypothesis being that of a chronic low grade inflammatory process [11]. A relation also seems to exist between the immunocompetency of the patient and the affliction from the plasma cell type, since it is observed mainly in immunosuppressed patients. The plasma cell type carries a poor prognosis, with a median survival of about 30 months [7]. The treatment of this type of disease is a combination of the use of steroids, rituximab, chemotherapeutic agents, and radiotherapy. Surgical excision aims mostly at the relief from symptoms rather than cure, whereas remission seems to be achieved in some cases, when the aforementioned modalities are combined with splenectomy [12].

In the case we present, the patient was asymptomatic and the mass was discovered as an incidental finding, characteristics that are typical of the hyaline-vascular type of the disease. An interesting aspect of the case we present is the synchronous presence of a hepatic lesion, which was also difficult to define preoperatively. Additionally, imaging studies and perioperative findings do not distinguish retroperitoneal Castleman's disease from malignant retroperitoneal tumors, particularly in the presence of a hepatic lesion. Gross inspection also fails to distinguish the lesion from a malignant neoplasm, because it may develop dense adhesions to adjacent organs [4, 13]. A question remains, however, as to how safe it is not to operate or at least obtain a biopsy from a lesion whose location is in favour of malignancy [14]. Retroperitoneal Castleman's disease should be added to the extensive list of differential diagnoses for tumors in the pararenal retroperitoneum [13]. Radical surgery can be avoided safely, given the excellent outcome after total resection of the tumor, whereas, even in the unifocal form of the disease, long-term followup is recommended, since the development of neoplasms such as Kaposi sarcoma has been reported as much as 8 years after the initial diagnosis of Castleman's disease [7].

## 4. Conclusions

Castleman's disease is a lymph node disease. Its multicentric form seems to be related with human herpes virus 8. It presents mainly in two types: the unifocal (hyaline vascular type) and the multifocal (plasma cell type). The localized hyaline vascular form presents as a solitary lesion which can be located mainly in the mediastinum, but also in the abdomen, retroperitoneum, or in many other locations. A good preoperative workup and an open biopsy during surgery, for an abdominal or retroperitoneal lesion if no definitive preoperative diagnosis has been established, can help to avoid extensive resection when dealing this benign disorder. Complete surgical excision is curative; recurrences have only been described after incomplete resection. The prognosis is excellent with a five years survival of nearly 100%.
